# International Delphi study on terminology, organisation and outcomes of geriatric rehabilitation for older people living at home

**DOI:** 10.1007/s41999-025-01241-9

**Published:** 2025-06-04

**Authors:** Astrid Preitschopf, Marisa Vaz, Marije Holstege, Margriet Pol, Bianca Buurman, Wim Groen

**Affiliations:** 1 Department of Medicine for Older People, Amsterdam UMC, Location Vrije Universiteit Amsterdam, de Boelelaan 1109, 1081 HV, Amsterdam, The Netherlands; 2 Aging & Later Life, Amsterdam Public Health, Amsterdam, The Netherlands; 3 Omring and Zorgcirkel, Department of Research GRZPLUS, GRZPLUS, Hoorn, The Netherlands; 4 Department of Research Omring, Omring, Hoorn, The Netherlands; 5 Research Group Geriatric Rehabilitation, Faculty of Health, Sports and Social Work, Centre of Expertise Prevention in Health and Social Care, Inholland University of Applied Sciences, Amsterdam, Netherlands; 6 Research Group Occupational Therapy: Technology and Participation, Faculty of Health, Centre of Expertise Urban Vitality, Amsterdam University of Applied Sciences, Amsterdam, The Netherlands; 7 Amsterdam Movement Sciences, Ageing & Vitality, Rehabilitation & Development, Amsterdam, The Netherlands

**Keywords:** Home-based, Geriatric rehabilitation, Delphi study, Older people

## Abstract

**Purpose:**

Internationally, many differences are observed regarding the practice of geriatric rehabilitation for older people living at home. To improve international collaboration and research on this topic, we aimed to reach a consensus on terminology, organisational aspects, and outcome domains to focus on.

**Methods:**

We conducted a three-round online Delphi study among 60 (Home-based) geriatric rehabilitation experts from 14 countries. In the first round, we collected diverse perspectives and input through statements and questions. In the subsequent rounds, participants rated statements on a 5-point Likert scale. Each statement could be accompanied by written feedback. After each round, results were presented anonymously to the participants, and statements on which no consensus was reached were rephrased. Consensus was defined as 70% or more participants (fully) agreeing with a statement.

**Results:**

Sixty, 52, and 46 experts completed rounds 1, 2 and 3, respectively. After two rounds, we reached a consensus on ten statements and on the remaining four in the last round. A consensus was reached on the terminology used (i.e., home-based geriatric rehabilitation) and on several organisational aspects (e.g., essential aspects to consider for starting home-based geriatric rehabilitation and the importance of a knowledgeable case manager). Lastly, experts agreed that participation and activity are the most important outcome domains to focus on.

**Conclusions:**

Through an international Delphi study, we reached consensus on various important aspects of home-based geriatric rehabilitation. These outcomes provide a basis for further development of this emerging field.

**Supplementary Information:**

The online version contains supplementary material available at 10.1007/s41999-025-01241-9.

## Key summary points

**Aim:**

To reach consensus on terminology, organisational aspects, and outcome domains of geriatric rehabilitation for older people living at home.

**Findings:**

In three rounds, an international panel reached a consensus regarding the term “home-based geriatric rehabilitation” to distinguish it from inpatient rehabilitation. The panel also identified key organisational aspects essential for its implementation and concluded that participation and activity are the primary outcome domains to focus on.

**Message:**

The results of this International Delphi shows consensus of experts on various topics in home-based GR, which is important to further develop international collaboration, development and research on this topic.

**Supplementary Information:**

The online version contains supplementary material available at 10.1007/s41999-025-01241-9.

## Introduction

Geriatric rehabilitation (GR) for older adults living at home has received increasing attention over the past years [1, 2]. Internationally, the delivery of GR in an outpatient setting, at home, or in the patient’s residence with a specialised team is referred to by various terminologies such as ambulatory rehabilitation, outpatient rehabilitation, or home-based rehabilitation [1, 3, 4]. Due to the lack of a universally accepted term, we use GR@Home throughout this study. This increasing attention for GR@Home is partly due to societal developments [5–7] and the preference of older adults to remain at home as long as possible [8]. Additionally, professionals prefer to offer rehabilitation in a structured, evidence-based way from an outpatient setting and wish GR@Home to be accessible to every patient [3, 9–11]. 

In a previous Delphi study conducted by Van Balen et al.[3], a consensus was reached on many aspects of GR despite significant variations in its organisation between countries [12]. These variations include the location of the GR department, such as in a hospital or a skilled nursing facility, and the composition of the multidisciplinary team. These differences are shaped by the healthcare system, as well as by applicable laws and regulations. However, the study of Van Balen et al. only touched on GR@Home briefly, leaving many questions unanswered. Therefore, further research on GR@Home as part of GR in total is necessary to understand its content and effective rehabilitation methods, including the preconditions for starting GR@Home, rehabilitation environment, interprofessional collaboration, and essential outcome domains to focus on [1, 3, 11–13].

Additionally, two recent studies on GR@Home [4, 9] demonstrated an overview of the “building blocks” of structure, process, environmental, and outcome components. This knowledge provides a foundation for further international consensus, development, and testing of the GR@Home trajectory in clinical practice. Nonetheless, several topics need more agreement. Consensus on GR@Home’s terminology and organisation of care pathways is crucial for fostering international collaboration and enhancing research on GR@Home.

Furthermore, it is essential to have a better understanding and international consensus on GR@Home outcomes to compare scientific studies more effectively. For example, a recent systematic review [4] showed that it is challenging to accurately identify studies due to various definitions of GR@Home and the various outcome measures used, complicating the pooling results for meta-analysis.

Therefore, the current Delphi study aims to reach an international consensus on aspects related to the following three topics concerning GR@Home: (1) Terminology, (2) Structural, process, and environmental elements, divided into four subtopics: (i) preconditions to start GR@Home, (ii) rehabilitation environment, (iii) use of eHealth, and (iv) coordination of rehabilitation. (3) Outcome domains on which GR@Home should be focused.

## Methods

### Study design

We performed an online Delphi study with three rounds which is known as an appropriate and recognised method to reach consensus on a topic [14–16]. Consensus, or “collective agreement,” is widely recognised as an effective method in cases where scientific evidence is lacking.

### Participants and recruitment

Participants were recruited from and through the European Geriatric Medicine Society—Special Interest Group on Geriatric Rehabilitation. We approached the group members to form an international group of experts in the field of GR@Home. The participating experts received an information letter from the Special Interest Group secretariat provided by the moderators (AP and MV). The letter explained the study and invited the members to participate. To reach a varied international and multidisciplinary group, we asked the members to forward the letter to potentially eligible colleagues from other disciplines.

The experts needed to fulfil the following criteria: (1) at least 2 years of experience with GR (preferably with GR@Home), (2) having good skills in written English, (3) must belong to one of the following professional groups: geriatrician, physician specialised in older adult care, nurse (practitioner), physiotherapist, occupational therapist, speech therapist, social work, dietitian, psychologist, scientific researcher.

For our Delphi study, we chose at least 2 years of experience with GR rather than 2 years in GR@Home. The follow-up GR after inpatient GR varies across countries. It can be a continuation of GR or be transferred to primary care. This broader criterion ensures that all participants have relevant and comparable experience in geriatric rehabilitation, regardless of national differences in healthcare systems and service delivery models.

When an expert expressed interest in participating, the Delphi moderators sent them additional information about the Delphi study’s procedures. The moderators did not inform participants about the identity of other participants.

### Delphi process

The Delphi process comprised three subsequent rounds, each involving an electronic survey using the Castor Electronic Data Capturing software. All materials and communications were in English. The two moderators (AP, MV) performed material preparation, survey distribution, data collection, and initial data analysis. The first questionnaire was sent in December 2023 (see Appendix 1), and the deadline to complete the third round was by the end of May 2024. A reminder email was sent to the participants who did not respond in time. We aimed to establish a consensus on aspects related to the three topics: (1) Terminology, (2) Structural, process, and environmental elements, and (3) Outcome domains.

Participants scored statements on a 5-point Likert scale ranging from (1) fully disagree, (2) disagree, (3) neutral, (4) agree to (5) fully agree, and they were allowed to provide a comment in free text to support their response to each statement. They could also provide additional comments on each topic if necessary. A consensus was reached when over 70% of the participants scored “agree” or “fully agree” on a statement [15].

The research team (AP, MV, MP, MH, WG) developed the statements and questions for round 1 based on their expertise, previously conducted studies, and literature review on the three topics mentioned in the introduction [4, 9]. Additionally, the Post-Acute Care Rehabilitation quality framework (11) was used to formulate the statements. It is in turn based on two widely used models within healthcare: the Structure, Process, and Outcome model of Donabedian [17] and the International Classification of Functioning Disability and Health model of the World Health Organization, which includes the patient-centred aspect of rehabilitation [18].

A physician assistant and occupational therapist involved in GR@Home in the Netherlands tested the questionnaire for round 1, which was subsequently revised for content, clarity, and layout based on their feedback. The first part of the survey began by collecting information on participants’ backgrounds and professional experience. This also served to verify the eligibility criteria for participation in the Delphi study. The second part consisted of thirteen statements and ten closed and open-ended questions designed to explore opinions on the three topics and formulate statements for the next round. In contrast to round 1, rounds 2 and 3 consisted of statements only.

After each round, the moderators (AP, MV) conducted an initial analysis of the results and reviewed participants’ comments, which were then shared and thoroughly discussed with the research team (AP, MV, MP, MH, WG). In subsequent rounds, we shared the previous round’s results with participants, including the original findings, anonymous comments, and a summary for each statement or question. Based on the participants’ feedback, the research team rephrased statements on which no consensus was reached. Statements on which a consensus was reached, were excluded from further rounds. The Delphi study was set to conclude after three rounds.

### Data analysis

Descriptive statistics (SPSS, version 28) were used to describe participant characteristics and the results of the questions and statements.

### Ethical Considerations

This study was approved by the Medical Ethics Committee of the University Medical Centre Amsterdam in The Netherlands (protocol ID 2023.0552). The study objectives were outlined to all participants, and their informed consent was obtained before the first Delphi round started.

## Results

### Participant characteristics

Table 1 shows the characteristics of the participants. The first round of the study involved 60 experts from fourteen different countries, of which most were from countries in Europe (*n* = 12), with additional participants from Australia (*n* = 1) and Canada (*n* = 1)). The experts had seven professions; the most represented were physiotherapists (*n* = 22; 37%) and geriatricians (*n* = 20; 33%). In the two subsequent rounds, *n* = 52; 87% and *n* = 46; 77% experts took part, with a continuing fairly even distribution across the 14 countries and seven professions. The median (IQR) experience with geriatric rehabilitation for the entire group was 10.5 (6–19) years. Forty-six (76%) professionals indicated experience delivering geriatric rehabilitation at home. The median (IQR) experience was 5.0 (3.8–14.3) years.

**Table 1:** Characteristics of participants

	Round 1	Round 2	Round 3
Profession within GR (*n* = 60)	*N* = 60 (100%)	*N* = 52 (86.7%)	*N* = 46 (76.7%)
Geriatrician	20 (33.3)	15 (28.8)	13 (28.3)
Nurse practitioner	2 (3.3)	1 (1.9)	2 (2.2)
Occupational therapist	5 (8.3)	5 (9.6)	5 (10.9)
Other	4 (6.7)	4 (7.7)	3 (6.5)
Physiotherapist	22 (36.7)	21 (40.4)	19 (41.3)
Researcher	4 (6.7)	3 (5.8)	2 (4.3)
Speech therapist	1 (1.7)	1 (1.9)	1 (2.2)
Physician specialised in older adult care	2 (3.3)	2 (3.8)	2 (4.3)
Country (*n* = 60)	*n* (%)	*n* (%)	*n* (%)
Australia	1 (1.7)	1 (1.9)	1 (2.2)
Belgium	3 (5.0)	3 (5.8)	3 (6.5)
Canada	3 (5.0)	2 (3.8)	2 (4.3)
Germany	3 (5.0)	3 (5.8)	3 (6.5)
Ireland	8 (13.3)	7 (13.5)	7 (15.2)
Netherlands	8 (13.3)	7 (13.5)	6 (13.0)
Portugal	4 (6.7)	4 (7.7)	3 (6.5)
Romania	6 (10.0)	4 (7.7)	3 (6.5)
Scotland	1 (1.7)	1 (1.7)	1 (2.2)
Spain	5 (8.3)	4 (7.7)	2 (4.3)
Sweden	3 (5.0)	3 (5.8)	3 (6.5)
Switzerland	2 (3.3)	2 (3.8)	2 (4.3)
Turkey	6 (10.0)	4 (7.7)	4 (8.7)
United Kingdom	7 (11.7)	7 (13.5)	6 (13.0)
Years of experience in GR (*n* = 60)	Median (IQR)		
Years of experience	10.5 (6.0–19.0)		
The organisation provides GR for older adults living at home (*n* = 60)	*n* (%)		
Yes	36 (60.0)		
No	24 (40.0)		
Experience delivering GR for older adults living at home (*n* = 60)	*n* (%)		
Yes	46 (76.7)		
No	14 (23.3)		
Years of experience delivering GR for older adults living at home (*n* = 46)^a^	Median (IQR)		
Years of experience	5 (3.8–14.3)		

^a^*n* = 46 as this is a follow-up question to the previous question: experience delivering GR

*GR* geriatric rehabilitation, *SD* standard deviation

### Delphi rounds

During the first round, 60 participants completed the survey. Out of the 26 statements and questions covering the 3 main topics, a consensus was reached on one statement. In the second round, 52 participants completed the survey with 14 statements related to the same three main topics. In this round, a consensus was reached on ten statements with agreement ranging from 73 to 92%, while no consensus was reached on four statements with agreement ranging from 61 to 65% (as shown in Tables 2 and 3). In the third and final round, 46 experts completed the survey containing four statements related to the first two topics. Consensus was reached on all statements, with agreement ranging from 72 to 87% (Table 2).

**Table 2:** Statements on which consensus was reached

Topic	Statement		Level of agreement (%)	Consensus reached in round
Terminology	1	The most appropriate terminology internationally to indicate that a study concerning GR delivered to older adults living at home is “Home-based geriatric rehabilitation.”	84.8%	3
Preconditions to start GR@Home	2	To start GR@Home, a social support system and a safe home environment (for patients and professionals) are important aspects to consider This is primary *in addition to the general basic referral factors for GR* (i.e., a clinical, patient-centred decision based on patient characteristics, individual rehabilitation needs, motivation, and rehabilitation potential)	78.8%^a^	2
	3	When GR is executed as an inpatient, GR should preferably be followed by a GR@Home trajectory provided that the patient is motivated and has ongoing rehabilitation goals	82.7%^a^	2
	4	GR@Home should be ended when the GR goals are reached	71.7%	3
Challenging rehabilitation environment	5	A challenging rehabilitation environment during GR@Home can be achieved by implementing a reablement approach while focusing on the patient’s participation goals	73.1%^a^	2
	6	The main reasons for providing GR@Home in an outpatient setting rather than at the patient’s residence are the need for specific training equipment, the absence of a safe environment, the lack of social support, and financial or reimbursement aspects	73.1%^a^	2
EHealth	7	Using a blended care approach in which eHealth is integrated into in-person treatment may improve the efficiency of GR@Home while maintaining high-quality care	87.0%	3
	8	When it is expected that eHealth can be utilized during GR@Home, it should already be introduced during inpatient GR so that the patient can use it optimally during GR@Home	88.5%	2
Coordination of rehabilitation	9	Case management is an important element for optimal coordination of GR@Home	86.5%	2
	10	When it is decided that a patient will follow a GR@Home trajectory, it should be clear as soon as possible who will fulfil the role of case manager	80.4%	3
	11	The role of a case manager during GR@Home can be best fulfilled by a person (likely a healthcare professional) who has the right competencies (e.g. communication, coordination skills, knowledge of geriatric rehabilitation) regardless of the specific rehabilitation discipline	8.5%	2
	12	For continuous optimal functioning and participation in daily life a good transition from GR@Home to community services is necessary	92.3%^a^	2
Outcome domains	13	The most important outcome domains to focus on during GR@Home are participation and activity	88.2%	2
	14	Further research is needed to reach international consensus on the outcome measure(s) used for the domain participation during GR@Home	86.3%	2

^a^In response to the comments, we made textual adjustments to this statement to improve readability while maintaining its original content

**Table 3:** Statements on which no consensus reached in round 2

Topic	Statement		Level of agreement (%)
Terminology	1	The most appropriate terminology to use internationally to indicate that a study concerning GR delivered to older adults living at home: “Home-based geriatric rehabilitation for older adults”	61.5%
Preconditions to start GR@Home	4	GR@Home should be ended when the GR goals are reached or when the patient is ready to maintain or further improve functioning in daily life (if needed supported by (in)formal community care)	63.5%
EHealth	7	Considering the shortage of workforce capacity and the growing number of older people, the use of eHealth is a very important part of the solution to make GR@Home future-proof	65.4%
Coordination of rehabilitation	10	Case management starts with the initiation of GR@Home and can be preferably carried out by a professional from primary care (provided that there is a good transition of care). Alternatively, a professional of the inpatient team can fulfil this role	65.4%

### Delphi topics

The statements divided over the three main topics underwent iterative development throughout the three Delphi rounds, with participants’ scores and comments playing a crucial role in revising them. The dynamic nature of this process will be illustrated based on the qualitative feedback received. Here, we present a selection of the qualitative feedback received on the three topics. With this overview, we do not claim to be exhaustive but rather highlight the interesting points raised by the experts and how they were dealt with by the moderating team.

#### Topic 1: terminology

We intended to reach a consensus on which terminology is appropriate to use internationally for GR delivered to older adults living at home. In round 1, a large proportion (*n* = 38; 63%) of participants agreed that no other terminology is needed to distinguish between GR for older adults who are clinically admitted and those who live at home. Additionally, there was no agreement on the most appropriate terminology to use for GR for older adults living at home. Although *n* = 16; 73% of the participants answered the question ‘what is the most appropriate terminology to use internationally for GR@Home’ that the addition of home-based is suitable:

‘I prefer the term home-based GR because this implies that, when necessary, you can still come to a location where therapy will be provided. I especially dislike Outpatient GR and Ambulatory GR, because it doesn’t specify enough that it is taking place in the home setting.’ (round 1, occupational therapist, The Netherlands)

Based on the scores and comments from round 1, the following question emerged: Is a separate name needed for different settings and target groups, or is it sufficient to add the setting to the existing terminology, such as “home-based”. In round 2, only 61.5% agreement was reached on the statement: “The most appropriate terminology to use internationally to indicate that a study concerning GR delivered to older adults living at home: home-based geriatric rehabilitation for older adults. However, there was a tendency to include “home-based” to differentiate settings. Some participants noted that the use of both “geriatric” and “older adults” is redundant and some did not like the term “geriatric”:

‘I still don’t agree with the term geriatric, and I feel if patients hear that terminology, it will have a counterproductive effect. I do think including the term home-based is important.’ (round 3, clinical nurse specialist gerontology, Ireland)

In round 3, the participants reached a consensus (84.8%) on the statement that “home-based geriatric rehabilitation” is the most appropriate terminology to indicate a study concerning GR delivered to older adults living at home.

#### Topic 2: structural, process, and environmental elements

##### Topic 2.1: preconditions to start GR@Home

Referral to GR, in general, should be a clinical, patient-centred decision based on patient characteristics, individual rehabilitation needs, motivation, and rehabilitation potential [3]. In round 1, the experts were asked if there were additional factors to consider that were explicitly related to GR@Home. They could separately rate six predefined factors (i.e. the presence of a social support system or formal care, a secure home environment, travel distance, availability of supportive eHealth services at home, and having the capacity to use supportive eHealth services at home). Furthermore, they could add additional factors if needed.

Only the presence of an adequate social support system and a safe home environment were identified as necessary preconditions (>70% agreement). The experts mentioned that attention should be given to all the mentioned factors, but they should not become referral limitations for (and hence reduced accessibility to) GR@Home. Further, they noted that some aspects could be selection criteria but also rehabilitation goals:

‘I tend to agree that these are important factors to consider for referral for GR, however the statement lacks the impact of reason for considering these factors. In other words, is this important for selection, is this the rehabilitation goal or something else?’ (round 2, Physician specialised in older adult care, The Netherlands)

In addition to factors to consider when starting GR@Home, the experts were also presented with reasons for stopping it. In round 1, the reason for stopping ‘Depending on the needs’ was selected most frequently (61.7%). The experts commented that GR@Home focuses on achieving stable functioning in daily life rather than just further improvement.

In the second round, the experts were asked to rate the statement whether GR@Home should end when the GR goals are achieved or when the patient is ready to maintain or further improve functioning in daily life, on which no consensus was reached. Some participants highlighted that rehabilitation should empower independent living, with goals to prevent decline and avoid readmissions.

Despite the consensus in the third round that GR@Home ends when GR goals are reached, participants expressed concerns about the person-centred nature of these goals, as these may change during rehabilitation, especially for older adults with frailty or co-morbidities:

‘… What we initially set as goals may be changed during rehabilitation. So, we usually end when the goals are reached. I miss the dynamic of “goal- changing” here in this statement. Just remember that the goals must always be defined broadly as participation goals…’ (round 3, geriatrician, Germany)

##### Topic 2.2: challenging rehabilitation environment during home-based GR

In the first round, a consensus (83.3%) was reached on the proposed statement that a challenging rehabilitation environment at home is essential. Participants were asked to score various elements that could promote this rehabilitation environment, and only “Incorporating a reablement approach” scored above 70%. In the second round, the experts agreed (73.1%) that implementing a reablement approach while focusing on the patient’s participation goals can lead to a challenging rehabilitation environment during GR@Home.

Key discussion points for this topic included defining the terms and integrating GR@Home into daily life. Several participants mentioned that the terms “challenging rehabilitation environment” and “reablement approach” were unclear and relatively new to them. In Round 2, the research team provided definitions for the terms used in this Delphi study (Appendix 2). Additionally, participants emphasised the importance of respecting a person’s home and individual needs and wishes during GR@Home:

‘Home rehabilitation should be integrated into everyday life, with important activities and functional activities. Meaningful goals and activities create motivation, not general exercises…… The home is the natural context, and the staff must have the ability to use the home as a rehabilitation arena, not to create something else.’ (round 1, researcher, Sweden)

##### Topic 2.3: use of eHealth

There was no consensus about eHealth being essential (round 1) or necessary (round 2) for making GR@Home future-proof in the context of the shortage of workforce and the growing number of older people. Participants acknowledged that eHealth might be part of the solution but emphasised that it is not the only remedy for the workforce challenges. Besides, they stated that eHealth applications should be used to supplement care and not replace it. Experts see significant barriers to eHealth use among this population, underscoring the need to develop appropriate applications for older people:

‘EHealth provides many solutions but also barriers, and it is important to acknowledge the meaning of in-person meetings during the rehabilitation process. Today, many older adults do not have access to or know how to use the technology. This creates risks rather than enablers. eHealth is important for the development and execution of GR@Home, but for the moment, it should be considered as an option, not a necessity.’ (round 1, occupational therapist, Sweden)

The importance of a personalized approach was emphasized, particularly regarding the eHealth literacy of both patients and healthcare professionals. Experts agreed that for eHealth to be effectively used during the GR@Home program, it should be already introduced during the inpatient GR phase. This may facilitate optimal utilization, allowing healthcare professionals to assess the older person’s ability and proficiency with eHealth tools.

Finally, a consensus (87.0%) was reached in the third round on the statement that using a “blended care” approach, where eHealth is integrated into in-person treatment, may improve the efficiency of GR@Home while maintaining high-quality care.

##### Topic 2.4: coordination of rehabilitation

This subtopic explored the importance of case management, the timing for involving a case manager, and the most suitable person to fulfil this role. Participants acknowledge that a case manager is important and can be helpful but do not consider it to be essential. The opinions on the timing and most suitable person were diverse.

In round one, most participants (53.7%) preferred a primary care case manager, while 29.6% favoured someone from the inpatient multidisciplinary team. Round 2 also revealed no consensus on the best choice. Some participants stated that primary care professionals should fulfil this role because they can recognise the needs and possibilities for GR@Home. Others indicated that an inpatient team member should fulfil this role (if providing care in the community) because they have better knowledge of the patient’s trajectory:

‘… I would argue it is beneficial to have separate case managers who can be specialists in the setting in which they work. The community setting has different demands than inpatient work. In addition, both areas require a lot of networking, which does not fully overlap.’ (round 1, physical therapist, United Kingdom)

It was mentioned that ideally, the same case manager should oversee both inpatient GR and GR@Home, although this is not always feasible. However, the experts emphasised the importance of good communication between the inpatient, GR@Home, and community teams. The significance of this communication became even clearer by the broad consensus (92.3%) the experts reached in the second round, on the statement that a good transition of care is necessary for achieving optimal functioning and participation in daily life for the patient.

Initially, the specific discipline for the case manager role was unclear. However, round 2 established that it should be fulfilled by someone with appropriate competencies in geriatric rehabilitation. Some participants suggested that a patient could even take on this role. Concerns were raised about whether “case management” is the right term to use and about the potential burden placed on the case manager as the primary contact for patients and caregivers.

In round 3, the focus shifted more toward when case management should begin. We excluded the specific setting in which a case manager should work as it seems more important that the case manager has the right skills and competencies. Ultimately, a consensus (80.4%) was reached that, once it is decided the patient will follow a GR@Home trajectory, it should be determined who will fulfil this role as soon as possible.

#### Topic 3: outcome domains

We aimed to identify the most essential outcome domains on which GR@Home should focus. For this purpose, we initially concentrated on the eighth outcome domains from the Post-Acute Care rehabilitation quality framework: (1) Body structures and function, (2) Functional capacity, activity, (3) Functional capacity, participation, (4) Psychosocial and behavioural, (5) Environmental context, (6) Patient and family/caregivers’ health-related quality of life, (7) Consumers’ experience, (8) Healthcare utilisation. In the first round, we distinguished between outcome domains for scientific research and those for measuring individual outcomes in daily clinical practice.

In Round 1, participants were asked to rank the eight outcome domains in order of importance. The participants (*n* = 59) noted that assessments should be both comprehensive and efficient, with the outcome domains comprising a mix of physical function and quality of life as the main priority. They also identified the absence of a domain addressing frailty. Interestingly, the participants ranked the outcome domains almost identically for both clinical practice and scientific research. The top-rated domains were participation, activity, body structure, health-related quality of life, and psychological and behavioural.

Based on this, we formulated two new statements for Round 2 (see Table 2), where participants reached a consensus (88.2%) that “participation” and “activity” are the most essential outcome domains to prioritize during GR@Home. A noteworthy comment was that other outcome domains, such as health service use, should not be ignored:

‘Healthcare utilisation received few votes but should be considered from the program evaluation aspect or demonstrating value to funders. Function and participation are key to patients and care providers, but preventing or postponing the use of more expensive services is key to funders.’ (round 2, physical therapist, Canada)

Finally, participants reached a consensus (86.3%) that further research is needed to establish an international agreement on the outcome measure(s) for the domain of participation during GR@Home.

## Discussion

This Delphi study provides valuable insights into important aspects of home-based rehabilitation. Using an online survey, a large group of experts from 14 countries and seven professions reached a consensus on key aspects of home-based GR: terminology, organisational aspects, and outcome domains. This is the first international consensus study focussing exclusively on home-based GR.

A significant outcome of this study is agreement to adopt the term home-based GR to distinguish this type of rehabilitation from inpatient GR. Home-based rehabilitation is recognised as an essential component of the comprehensive GR care pathway [3] and requires tailored organisational aspects, making this distinction important. A uniform definition facilitates international collaboration and enhances the quality of care by providing patients with clearer information about their rehabilitation process, especially from their perspective [9, 19]. However, the term “geriatric” seemed to spark discussion about the suitability during all three Delphi rounds. This topic was considered outside the scope of our study, as we aligned with the terminology used by van Balen et al. [3]. Although we did not aim to evaluate the suitability of the term “geriatric” for the target group internationally, we recognize the importance of this debate.

This study reached a consensus on four sub-topics related to organisational aspects. First, experts agreed that a safe home environment and a strong social support system are essential preconditions for initiating home-based goal-related rehabilitation. These factors should be included as standard triage items and can also serve as inpatient goals to facilitate home-based GR [13]. Yet, experts’ comments highlighted international differences in how these preconditions are addressed, with various factors, such as cultural and infrastructural aspects, influencing these considerations [20]. Additionally, participants expressed confusion about the term “safe home environment” questioning whether “safe” refers to the patient’s safety, such as fall prevention, or the professional’s safety during home visits. In our opinion, “safe” refers to both aspects. Home-based GR can promote a safe home situation by allowing professionals to suggest necessary adjustments and identify which activities need to be practiced [18, 21, 22]. Additionally, professionals need a secure work environment while ensuring patient safety [22]. Furthermore, involving informal carers, who can be trained to support the patient, is also vital in creating a safe home environment [18, 23–25].

Secondly, in addition to safety, experts highlighted the importance of a challenging rehabilitation environment, advocating for a reablement approach focused on participation goals. While reablement is well-established in some countries, it was a relatively new concept for several participants. It is a person-centred, holistic model that, like geriatric rehabilitation, aims to promote independence in older adults. Its short-term, home-based nature and strong focus on individual goals align well with home-based GR, which likewise supports participation within the home environment [26, 27]. The concept of a challenging rehabilitation environment also raised questions among the expert panel and required additional clarification. According to Ramsey et al. rehabilitation at home promotes a stimulating environment because it encourages people to take up activities in daily life earlier [28]. Tijssen et al. also studied the concept of rehabilitation environment and developed a conceptual framework with five clusters to help create a challenging rehabilitation environment. This framework is primarily focussed on inpatient GR but could also be applicable to home-based GR [25, 29] since it highlights the active role of patients and their caregivers in stimulating practice during the entire rehabilitation process.

Thirdly, our Delphi study indicates consensus for initiating eHealth use in the inpatient setting (88.5%) and using it as a blended approach (87%) which is in line with Kraaijkamp et al. [30]. Research [31] demonstrated that the use of technology is not yet fully integrated into GR. For example, only half of their participants use eHealth (such as mobile apps and video consultations), of which 20% also combine it with in-person therapy. Successful implementation requires a clear definition of eHealth in GR, professional support, a clear organisational- and implementation strategy, user-friendly tools [31–33], and awareness of the positive and negative impact of using eHealth at home [34].

The final organisational aspect concerns the coordination of rehabilitation. Effective collaboration between inpatient GR professionals and community care providers is essential to ensure a smooth transition home for the patient [35]. A skilled case manager can play a pivotal role in facilitating this process and may offer significant added value. However, this role needs further development within home-based GR. We could learn from other fields, like transitional care, where effective care coordination has been shown to reduce readmission rates [36]. Given the fragmented nature of the healthcare system and the changing care needs across different stages of rehabilitation[37], continuity of care is critical [35, 38–40]. Therefore, strengthening collaboration across care settings is key to delivering efficient and effective rehabilitation [35]. 

Regarding outcome domains, experts identified participation as the key outcome domain in home-based GR, as the main goal of GR is to restore functioning and levels of participation. However, the focus often remains on the biomedical model, which results in limited attention to participation and its infrequent use as an outcome domain [4, 30, 41]. Our study shows some agreement on outcome measures; however, the existence of numerous overlapping measurement instruments for each outcome domain complicates their implementation [42].

### Strengths and limitations

A strength of this study is its collaboration with the Special Interest Group GR of the European Geriatric Medicine Society. The diverse group of experts were recruited from and through the Special Interest Group GR, including professionals and researchers with experience in (home-Based) GR, and provided valuable practical and scientific insight. Another strength is the relatively high retention of participation across all three rounds, which helps to minimise bias from selective dropout.

Although 14 countries participated in this Delphi study, this represents a relatively small proportion on a global scale, which may have affected the generalizability of the findings. While the participant pool may be limited globally, a significant advantage is that the participants are connected to the Special Interest Group and engaged in development and international coordination.

Another limitation is the overrepresentation of geriatricians and physiotherapists among the participants. Despite efforts to include individuals from various disciplines, recruiting more participants from fields such as (community) nursing, speech therapy, and dietetics would have been beneficial. Furthermore, this study did not involve all stakeholder groups, such as patients and informal caregivers. This decision was intentional, as including them would have required a different approach, language, and questionnaire. However, it is worth noting that their perspectives are indirectly reflected in the knowledge gathered from various qualitative studies [9, 19], which were incorporated in this study.

Finally, we acknowledge that the topics discussed represent a selective sample shaped by international differences in the organisation of home-based GR and the varying healthcare laws across countries that influence its organisation and content. To address these variations, we adopted a comprehensive approach that integrated multiple sources of evidence including a systematic review with meta-analyses [4], and qualitative studies [9, 19], to identify relevant topics. Additionally, our research group included experts in home-based GR with substantial experience in both daily practice and research, which enhanced the identification of subjects and the subsequent analysis of the results.

### Implications for practice and research

This study has initiated a discussion and raised awareness about home-based GR. Several elements are essential for practice, considering necessary adaptations needed in the local context: These include: (1) a blended care approach; integrating eHealth into personal treatments, can improve the efficiency of home-based GR while maintaining the quality of care, (2) Timely introduction of eHealth; eHealth should be introduced during inpatient GR, to ensure that the patient can use it optimally during home-based GR, (3) Case management; effective coordination of home-based GR is essential. It is important to clarify, as early as possible, who will fulfil this role, (4) Transition to community services: Ensuring a smooth transition from home-based GR to community services is necessary for continued optimal functioning and participation in daily life.

In addition, further research is recommended in the following areas: (1) The appropriateness of the term “geriatric” in this context (and potentially in general) should be (re)evaluated, (2) Refining of the concept “challenging rehabilitation environment” to enable its effective application in the home setting, (3) The organisation and implementation of eHealth tools to optimise the effectiveness of home-based, (4) Strengthening the collaboration between inpatient GR professional and community care professionals, (5) The potential benefits of a case manager’s role in coordinating home-based GR. (6) The establishment of a core set of measurements specifically for the GR trajectory including both inpatient and home-based care. (7) Reaching international consensus on the outcome measures used for the participation domain during home-based GR.

## Conclusion

This Delphi study investigated the terminology, organisational aspects, and outcome domains of home-based GR, achieving consensus among experts on its key elements. The term “home-based GR” was agreed upon to clearly define the distinct nature of rehabilitation delivered in the home setting. The study identifies critical factors essential for effective rehabilitation, including a safe home environment and strong social support. It also emphasizes the importance of creating a challenging rehabilitation environment that encourages patient participation. Additionally, the study underscores the potential role of skilled case managers in coordinating care. Finally, it highlights participation as a primary goal of home-based GR. Overall, this study lays the groundwork for improved practices in home-based GR and encourages future research and international collaboration to enhance care for older adults. 

## Supplementary Information

Below is the link to the electronic supplementary material.

Supplementary file1 (DOCX 86 KB)

## Data Availability

Data will be made available, from the corresponding author on reasonable request.
