# Supplementary material for: International Delphi study on terminology, organisation and outcomes of geriatric rehabilitation for older people living at home
Source: Eur Geriatr Med. 2025 Jun 4;16(5):1839–50. doi: 10.1007/s41999-025-01241-9 (PMC12528263; doi:10.1007/s41999-025-01241-9)
Supplement: Supplementary file 1 — Supplementary file1 (DOCX 86 KB) [file 41999_2025_1241_MOESM1_ESM.docx]

Supplementary material

**Title:** International Delphi Study on Terminology, Organisation and Outcomes of Geriatric Rehabilitation for Older People Living at Home

**Journal name**: European Geriatric Medicine

**Author names:** Astrid Preitschopf*^,^ Marisa Vaz*, Marije Holstege, Margriet Pol, Bianca Buurman, Wim Groen

* Shared first author

**Affiliation and e-mail address of the corresponding author:** Astrid Preitschopf***^1,2,3^***

(1)Amsterdam UMC, location Vrije Universiteit Amsterdam, Department of Medicine for Older People, Amsterdam, The Netherlands
(2) Amsterdam Public Health, Aging & Later Life, Amsterdam, The Netherlands
(3) GRZPLUS; Omring and Zorgcirkel, Department of Research GRZPLUS, Hoorn, The Netherlands
E-mail: [a.d.vangestel@amsterdamumc.nl](mailto:a.d.vangestel@amsterdamumc.nl)

Content

[**Appendix 1 Survey Round 1** 2](#_Toc190938115)

[General questions 2](#_Toc190938116)

[Topic 1: Terminology 2](#_Toc190938117)

[Topic 2: Structure, process, and environment elements 3](#_Toc190938118)

[Topic 2.1: Preconditions to start GR@Home 3](#_Toc190938119)

[Topic 2.2: Challenging Rehabilitation environment during GR@Home. 4](#_Toc190938120)

[Topic 2.3: Use of eHealth 5](#_Toc190938121)

[Topic 2.4: Coordination of rehabilitation (case manager) 5](#_Toc190938122)

[Topic 3: Outcome Domains 6](#_Toc190938123)

[References 7](#_Toc190938124)

[**Appendix 2 Clarification of terms used in this Delphi** 9](#_Toc190938125)

# Appendix 1 Survey Round 1

## General questions

1. What is your profession within Geriatric Rehabilitation (GR): Doctor/ geriatrician/specialist elderly care, nurse practitioner, Inpatient nurse, Community care nurse, Physiotherapist, Occupational therapist, Speech therapist, Dietician, Psychologist, Social worker, Manager/team leader, Researcher, Other. Please fill in the open text field
2. How many years of experience do you have in Geriatric Rehabilitation --------- years

- Lower limit two years

1. In which country do you practice Geriatric Rehabilitation?
2. Does your organization provide Geriatric Rehabilitation for older adults living at home?
3. Do you have experience delivering Geriatric Rehabilitation for older adults living at home?
4. How many years of experience do you have with delivering Geriatric Rehabilitation for older adults living at home? --------- years

## Topic 1: Terminology

**Introduction**

Geriatric Rehabilitation (GR) is defined as “*a multidimensional approach of diagnostic and therapeutic interventions to optimize functional capacity, promote activity, and preserve functional reserve and social participation in older people with disabling impairments”(10).*  Internationally, there are different terminologies used for GR delivered to older adults living at home, and it can be executed in different settings, such as at the patient’s residence or a centre specialized in GR.

With the following questions, we want to reach a consensus on **which** terminology is appropriate to use internationally. Please rate the following statement. If the score is equal to or lower than three, the question arises of what other Terminology is appropriate. You can use the open text field to add a comment explaining the argument(s) for your response.

1. The Terminology “Geriatric Rehabilitation” should be the same for older adults who are clinically admitted and those who live at home. No different terminology is needed to distinguish between these two settings. If you select 1,2 or 3 on the Likert scale a follow up question will arise.
2. Please select the most appropriate terminology to use internationally for GR@Home.
   1. Outpatient Geriatric Rehabilitation
   2. Ambulatory Geriatric Rehabilitation
   3. Home-Based Rehabilitation
   4. Geriatric Home-Based Rehabilitation
   5. Home-based Geriatric Rehabilitation
   6. Other, namely (use the open text field to fill in your suggestion)
3. Do you have any further comments regarding the terminology of Geriatric Rehabilitation delivered to older adults at home?

## Topic 2: Structure, process, and environment elements

**Introduction**

Preitschopf et al. (7) demonstrated in a systematic review various frequently used structural, procedural, and environmental elements of GR@Home based on the Post-Acute Care Rehabilitation (PAC-Rehab) quality framework by Jesus et al. (11). You can find more information on this framework via this [link](<https://doi.org/10.1093%2Fageing%2Fafac300>).

In addition to the frequently used elements, many differences were found between the studies. Some organizational elements were mentioned sporadically, such as using eHealth technology. In addition, there were many differences in the preconditions to start GR@Home, the duration of the GR@Home trajectory, and the rehabilitation environment. The study concluded that there is no consensus about the organization and content of a GR@Home trajectory.

The following statements concern preconditions to start GR@Home, rehabilitation environment, use of eHealth, and rehabilitation coordination (case manager).

### Topic 2.1: Preconditions to start GR@Home

In the Delphi study of van Balen et al (5), a consensus was reached that: *“Ambulatory GR should be available for patients immediately following hospital discharge, and Referral to ambulatory GR should be available for patients living in the community and a hospital/institutional admission before starting GR should not be necessary”.*

Also, a consensus was reached that *“referral to GR should be a clinical, patient-centred decision based on patient characteristics, individual rehabilitation needs, motivation, and rehabilitation potential*”.

However, are these factors sufficient to refer to GR@Home or are there additional factors to consider?

1. Referral to GR@Home is based on the **exact** same factors as referral to GR in general. It should be a clinical, patient-centred decision based on patient characteristics, individual rehabilitation needs, motivation, and rehabilitation potential.

Referral to GR@Home might be based on more or different factors than referral to inpatient GR like the patient characteristics, individual rehabilitation needs, motivation, or rehabilitation potential.

**On which factors should the decision additionally be based?** Please review the following six factors and evaluate them on a Likert scale, ranging from full disagreement to full agreement.

1. The presence of a social support system.
2. The presence of formal care.
3. A secure home environment, in general.
4. The travel distance between the rehabilitation center and the patient's home and vice versa.
5. The availability of older adults to use supportive eHealth services at home.
6. The capacity of the older adult to use supportive eHealth services at home.
7. Are there any other additional factors to consider? Please provide your input in the comment field.

Van Balen et al. (5) found that Geriatric Rehabilitation should be preferably executed in an ambulatory setting, preferably in the patient's own home, and supported by a dedicated multidisciplinary team. Can we state the following?

When GR is executed inpatient, GR should always be followed by a GR@Home trajectory.

1. GR@Home should end when; (select one or more items):
   1. No further improvement can be made regarding the set goals for the GR trajectory, depending on patients’ needs.
   2. A multidisciplinary team specialized in GR is not needed for the treatment anymore.
   3. The trajectory, based on the individualized goals, with a pre-agreed duration has finished.
   4. Other, please fill in the comment field.

### Topic 2.2: Challenging Rehabilitation environment during GR@Home.

In the Delphi study from van Balen et al. (5) consensus was reached that “*GR should be preferably executed in an ambulatory setting, preferably in the patient’s own home, and supported by a dedicated team*”, and that “*the multidisciplinary team, especially the nursing staff, should establish a therapeutic climate on the GR ward.”*

Preitschopf et. al., performed a qualitative study (article under submission) in which the patient, healthcare professional, and organizational perspectives on GR@Home are collected and analysed. This study demonstrates that the situation at home is different from inpatient GR. Most patients find it difficult to ask for help and support, and on the other hand, they lack the support and external motivation to do the (homework) exercises. Additionally, professionals are not always able to implement an environment that simulates inpatient rehabilitation at home, the so-called rehabilitation environment at home. The next questions concern the rehabilitation environment.

1. Is it important to promote a challenging rehabilitation environment at home? If you select yes, the second question will arise. When you choose no, you will go directly to the next item.

- Yes/no (Yes --> statement 15, No --> statement 16)

1. To promote a challenging rehabilitation environment at home the following elements are important. Choose one or more items.
2. A focus on participation goals.
3. Support by an informal caregiver.
4. Educated informal caregivers on the rehabilitation environment.
5. Educated primary care healthcare professionals on the rehabilitation environment.
6. Training modules specific for training in the home environment.
7. The availability of training equipment at home.
8. Using eHealth for monitoring, training, coaching, and/or promoting self-management. The World Health Organization defines eHealth as the cost-effective and secure use of information and communications technologies in support of health and health-related fields, including healthcare services, health surveillance, health literature, and health education, knowledge, and research.
9. Incorporating a reablement approach. Reablement is a person-centered approach that helps individuals to learn or re-learn the skills necessary to be able to engage in activities/occupations that are important to them.
10. Other, please fill in the comment field.
11. A consensus is reached that GR should be preferably executed in an ambulatory setting at the patient's residence (5). However, in daily practice, it is not always possible to provide therapy at home. Identify one or more reasons to provide therapy in the clinical setting during GR@Home.
12. Access to specific training equipment.
13. Support from social interactions with other older adults in GR.
14. No safe environment at home
15. In the context of efficiency: for example, group training, time management, and agenda planning.
16. The financial aspects and insurance policies regarding non-comprehensive coverage (e.g., (reimbursement of) travel expenses, transportation).
17. There are no factors.
18. Other factors to take into consideration, please fill in the open field.

### Topic 2.3: Use of eHealth

WHO defines eHealth as the cost-effective and secure use of information and communications technologies in support of health and health-related fields, including healthcare services, health surveillance, health literature, and health education, knowledge, and research.

In the Delphi study from van Balen et al. (5), consensus was reached that “*Development and implementation of eHealth and technology are important and should be underway in the organization of geriatric rehabilitation.”* And *“eHealth should be used to facilitate the transition from inpatient to ambulatory GR and uniform recording throughout the total GR pathway."*

We here present statements regarding the use of eHealth during GR@Home:

1. The use of eHealth is **essential** to make GR@Home future-proof.
2. EHealth should already be introduced during inpatient GR so that the patient becomes familiar with it and it can be used optimally during GR@Home.

### Topic 2.4: Coordination of rehabilitation (case manager)

Previous research demonstrated important elements associated with the organization and content of a GR@Home trajectory, such as a case manager, a dedicated specialized team, caregiver involvement from admission to GR, and integrated care (6, 7, 12-14).

In the Delphi of van Balen et. al. (5), the consensus is reached: "If possible, to maintain continuity of care, the same GR team should supervise rehabilitation in both the institutional and community setting". The leader of the team should be the physician. Participants of that Delphi study stressed that the person in the lead depends on the leading skills and not what is written in the job title.

The next statement and questions concern the coordination of rehabilitation (case manager).

1. A case manager is essential for the coordination of GR@Home. If you select 3, 4 or five, the next questions concerning the case manager will arise. When you choose 1 or 2, you will go directly to the next item.
2. The same case manager during inpatient GR and GR@Home is essential for the continuity of rehabilitation.

The case manager should be someone from

Primary/community care**,** who will be consulted during inpatient GR and will become the person in the lead during the GR@Home trajectory.

The inpatient multidisciplinary team and guide the patient during the complete GR trajectory.

Other, please fill in the open text field to leave your comment.

1. The role of a case manager can best be fulfilled by a:
   1. Community nurse
   2. Ward nurse
   3. General practitioner
   4. Elderly care physician, specialist in GR
   5. Physiotherapist
   6. Occupational therapist
   7. Dietician
   8. Psychologist
   9. Social worker
   10. The case manager is a specific and separate discipline within the multidisciplinary team.
   11. Other, please fill in the open text field to leave your comment.
2. Do you have any further comments regarding the topic structure, process, and environment?

## Topic 3: Outcome Domains

**Introduction**

The review by Preitschopf et al.(7), showed that many different measurement instruments are currently used internationally, which hampers comparison and pooling of studies in meta-analyses. To improve the opportunities for comparison between international studies on GR@Home, the opportunities for international collaborative research, and the opportunities for the exchange of best practices, international consensus on used measurements is needed. Furthermore, in the review of Preitschopf, it was remarkable that participation was only used as an outcome in 30% of the studies, although the main goal of geriatric rehabilitation is to restore functioning and participation levels. Additionally, the qualitative study of Preitschopf et al. (under submission) found that patients, professionals, and policymakers are all focused on regaining patient autonomy and improving participation.

In the Delphi study of van Balen et al. (5), consensus was reached *that multidisciplinary general measurement/assessment instruments (feasible for every GR patient) should be used for the individual evaluation of patient’s functioning and participation, for benchmark and scientific research.*

In this Delphi study, we want to identify the most important outcome domains on which GR@Home should be focused. We distinguish between **outcome domains to conduct scientific research on GR@Home** and **domains to measure individual outcomes of GR@Home in daily clinical practice**.

We use the Post-Acute Care Rehabilitation (PAC-Rehab) quality framework (11) to classify the different outcome domains. (Click [here](<https://doi.org/10.1016/j.apmr.2014.12.007>) for more information on this framework.)

The different outcome domains are:

1. Body structure and function
2. Functional capacity, activity
3. Functional capacity, participation
4. Psychosocial and behavioural
5. Environmental context
6. Patient & family/caregivers Health-Related Quality of Life (HRQoL)
7. Consumers experience
8. Healthcare utilization

In your expert opinion, please rank the eight outcome domains in order of importance.

1. Ranking of the outcome domains in the context of scientific research on GR@Home.
2. Ranking of the outcome domains in the context of measuring individual outcomes on GR@Home in daily clinical practice.
3. Do you have any further comments regarding outcome domains on which GR@Home should be focused?

## References

1. Kenneth Gopal JdK, Dana Linckens, Bert Marchal, Annelies Veen. Sustainability of elderly care until 2050. Scenarios for future healthcare use, labor market and housing. Netherlands: ABF research; 2022 04-01-2022.

2. Jones M, Collier G, Reinkensmeyer DJ, DeRuyter F, Dzivak J, Zondervan D, Morris J. Big Data Analytics and Sensor-Enhanced Activity Management to Improve Effectiveness and Efficiency of Outpatient Medical Rehabilitation. Int J Environ Res Public Health. 2020;17(3).

3. Health WGsoaa. UN Decade of Healthy Ageing: Plan of Action 2021-2030. 2020.

4. Hatcher D, Chang E, Schmied V, Garrido S. Exploring the Perspectives of Older People on the Concept of Home. J Aging Res. 2019;2019:2679680.

5. van Balen R, Gordon AL, Schols J, Drewes YM, Achterberg WP. What is geriatric rehabilitation and how should it be organized? A Delphi study aimed at reaching European consensus. Eur Geriatr Med. 2019;10(6):977-87.

6. Becker C, Achterberg W. Quo vadis geriatric rehabilitation? Age Ageing. 2022;51(6).

7. Preitschopf A, Holstege M, Ligthart A, Groen W, Burchell G, Pol M, Buurman B. Effectiveness of outpatient geriatric rehabilitation after inpatient geriatric rehabilitation or hospitalisation: a systematic review and meta-analysis. Age Ageing. 2023;52(1).

8. ActiZ. The signals on green for rehabilitation in the home situation 2023. Available from: [Actiz](<https://www.actiz.nl/de-seinen-op-groen-voor-revalidatie-de-thuissituatie>).

9. Q-consultzorg. Reporting assessment of care content geriatric rehabilitation. [In Dutch] The Netherlands: Q-conslut; 2019 30-01-2019.

10. Grund S, Gordon AL, van Balen R, Bachmann S, Cherubini A, Landi F, et al. European consensus on core principles and future priorities for geriatric rehabilitation: consensus statement. Eur Geriatr Med. 2020;11(2):233-8.

11. Jesus TS, Hoenig H. Postacute rehabilitation quality of care: toward a shared conceptual framework. Arch Phys Med Rehabil. 2015;96(5):960-9.

12. Feng W, Yu H, Wang J, Xia J. Application effect of the hospital-community integrated service model in home rehabilitation of stroke in disabled elderly: a randomised trial. Ann Palliat Med. 2021;10(4):4670-7.

13. Holstege MS, Caljouw MA, Zekveld IG, van Balen R, de Groot AJ, van Haastregt JC, et al. Changes in geriatric rehabilitation: a national programme to improve quality of care. The Synergy and Innovation in Geriatric Rehabilitation study. Int J Integr Care. 2015;15:e045.

14. Prins LAP, Gamble CJ, van Dam van Isselt EF, Stammen RAI, Ettaibi A, Creemers IAM, van Haastregt JCM. An Exploratory Study Investigating Factors Influencing the Outpatient Delivery of Geriatric Rehabilitation. Journal of Clinical Medicine. 2023;12(15):5045.

# **Appendix 2 Clarification of terms used in this Delphi**

| Challenging environment | By challenging environment, we mean that patients are in an environment where they will be challenged to be active and participate in daily activities, doing (homework) exercises, and self-management is stimulated. |
| --- | --- |
| EHealth | The WHO defines eHealth as the cost-effective and secure use of information and communications technologies supporting health and health-related fields, including healthcare services, health surveillance, health literature, and health education, knowledge, and research. |
| Reablement | Reablement is a person-centred approach that helps individuals learn or re-learn the skills necessary to engage in activities/occupations that are important to them. Key elements within this approach are stimulating self-management, supporting informal caregivers, and involvement of the (in)formal social network. |
| Safe home environment | By a safe home environment, we mean a home in which the patient can move safely (wheelchair-bound or not), where necessary adjustments and aids are available, and where a safe working environment for professionals can be guaranteed. |
| Social support system | With a social support system, we mean informal social support such as family, friends, and neighbours. |
